# Molecular and cellular mechanisms of the age-dependency of opioid analgesia and tolerance

**DOI:** 10.1186/1744-8069-8-38

**Published:** 2012-05-21

**Authors:** Jing Zhao, Xin Xin, Guo-xi Xie, Pamela Pierce Palmer, Yu-guang Huang

**Affiliations:** 1 Department of Anesthesia, Peking Union Medical College Hospital, Chinese Academy of Medical Sciences & Peking Union Medical College, Beijing, 100730, China; 2 Department of Anesthesiology and Perioperative Care, University of California, San Francisco, CA, 94143, USA; 3 Department of Anesthesiology and Perioperative Care, University of California, San Francisco, CA, 94143, USA; 4 Department of Anesthesia, Peking Union Medical College Hospital, Chinese Academy of Medical Sciences, No.1 Shuaifuyuan Str., Wangfujing Ave., Dongcheng District, Beijing, 100730, China

**Keywords:** Molecular and cellular mechanisms, Age-dependency, Opioid tolerance

## Abstract

The age-dependency of opioid analgesia and tolerance has been noticed in both clinical observation and laboratory studies. Evidence shows that many molecular and cellular events that play essential roles in opioid analgesia and tolerance are actually age-dependent. For example, the expression and functions of endogenous opioid peptides, multiple types of opioid receptors, G protein subunits that couple to opioid receptors, and regulators of G protein signaling (RGS proteins) change with development and age. Other signaling systems that are critical to opioid tolerance development, such as N-methyl-D-aspartic acid (NMDA) receptors, also undergo age-related changes. It is plausible that the age-dependent expression and functions of molecules within and related to the opioid signaling pathways, as well as age-dependent cellular activity such as agonist-induced opioid receptor internalization and desensitization, eventually lead to significant age-dependent changes in opioid analgesia and tolerance development.

## Background

Opioid drugs, such as morphine, are commonly used analgesics that are effective for treating most acute and chronic pain conditions. However, prolonged and repetitive opioid treatment can have side effects and result in a significant reduction or even complete loss of the analgesic effect (i.e. tolerance). Thus, although opioid analgesia remains a powerful means of pain therapy, opioid tolerance has become a major clinical problem for many patients who receive daily opioids for pain conditions. Tolerance is also a long-standing problem in the basic pharmacology of opioids. Opioid drug research and development has yet to produce potent and type-selective opioid agents that do not cause tolerance because we still have a poor understanding of the mechanisms by which opioid analgesia occurs and opioid tolerance develops.

The mechanisms of opioid analgesia and tolerance are complicated, involving numerous molecules and cells, as well as many reactions and processes. These mechanisms act in concert across multiple levels: molecular, cellular, neuronal interaction and network, hormonal, and systemic. In addition, several physiological factors, such as age, sex, and genetic variations, can directly or indirectly affect the analgesic effectiveness and tolerance development of opioid drugs.

Recently, age-specific opioid therapy for pain, as well as the relationship between opioid tolerance and aging, has drawn considerable attention and renewed interest. It is well known that age-related processes (including early development and aging) play essential roles in the expression and function of many genes and in the development and function of many cells, tissues, and whole organisms. Studies also suggest that age is a determinant of opioid analgesia and tolerance in human beings, animals, and individual cells. The bases of such age-dependency are as complicated and poorly understood as the mechanisms of opioid analgesia and tolerance themselves, and they involve multiple levels.

This review discusses recent evidence supporting the concept that opioid analgesia and tolerance are age-dependent. It also explores the molecular and cellular mechanisms that underlie this phenomenon.

## Age-dependent opioid analgesia and tolerance

Results from many clinical observations and laboratory studies strongly support the notion that age is an important factor affecting opioid analgesia. Recent clinical and laboratory data indicate that age also affects the development rate of opioid tolerance.

### Age-dependent opioid analgesia and tolerance in patients with pain

For many years, studies have shown that age significantly influences the dosing and analgesic effects of commonly used opioid drugs [1,2]. In recent years, there has been a significant increase in the use of daily opioids to treat chronic nonmalignant pain [3]. There continues to be some debate as to whether daily opioid use results in sustainable pain relief for chronic nonmalignant pain conditions [4]. Clinical studies of long-term opioid administration show that although it can produce prolonged pain relief in many patients, some patients with nonmalignant pain require escalating doses of opioids over time to maintain opioid efficacy [5,6], suggesting that opioid tolerance development is indeed a clinical problem.

Opioid escalation can occur for a variety of reasons, including underlying disease progression, addiction, and pharmacologic tolerance. There are diagnostic tools to identify disease progression, and there are guidelines to identify and manage pain patients who might be drug-seeking or have a history of substance abuse [7]. However, there are not yet any guidelines to identify patients who may be poor candidates for long-term opioid treatment because they are prone to rapid opioid tolerance development that would make long-term pain relief unsustainable. Furthermore, there are currently no drugs available to delay opioid tolerance. Therefore, the problem is two-fold. We need to identify important clinical parameters that affect opioid tolerance development and, in turn, find ways to use this information to pinpoint effective therapeutic targets for future drug development. Age could be an important member of this set of parameters.

It is widely observed in pain management that younger patients seem to develop opioid tolerance more rapidly than older patients. Although there have been numerous studies of the effects of age on the pharmacokinetics of opioids, potential age-related changes in clinical pharmacodynamic tolerance to long-term opioids has never been studied. Rather, the majority of clinical studies have examined patients aged 18–80 years as a single group, with the mean age usually in the 50- to 60-year range.

To determine whether opioid dose escalation and long-term pain relief with extended opioid treatment differs significantly among patients in different age groups, a recent retrospective study examined patients treated for an extended period with long-acting opioids for nonmalignant pain [8]. Opioid dose escalation and visual analog scale (VAS) scores were compared between patients less than 50 years old and patients older than 60 years. Significant differences were found; older patients, regardless of gender or type of pain, escalated their opioid use significantly less than younger patients over the 2-year treatment period.

A study by Moulin et al [9] showed that a group of 46 patients with chronic nonneuropathic pain and an average age of 40 years had to take up to 60 mg oral morphine twice a day to get sustainable pain relief. These doses were much higher than those used in patients aged 60 years and older.

### Age-dependent opioid analgesia and tolerance in laboratory animals

Rats of varying ages have been evaluated in single-dose studies to determine the effects of age on opioid pharmacokinetics and analgesic efficacy [10]. There also have been studies of opioid tolerance in prenatal and early postnatal animals [11-13]. Both types of studies have suggested that opioid tolerance is age-dependent, yet no study has systematically evaluated the rate of opioid tolerance development across an animal’s lifespan, from early adolescence to advanced maturity. An early study suggested that morphine tolerance after repeated daily administration occurred more rapidly in young rats; however, the oldest rats used in that study were 12 weeks old [14]. In a study of daily morphine administration in rats ranging in age from 3 weeks to 1 year, the time to onset of tolerance increased dramatically as the rats aged. This effect could not be explained by age-related changes in the pharmacokinetics of morphine, suggesting that cellular and molecular mechanisms of opioid receptor signal transduction may be involved [15].

Conflicting evidence exists concerning whether tolerance develops to opiate-induced antinociception during the first 2 postnatal weeks. Tolerance to the antinociceptive effects of morphine does develop in rats within 15 days after birth, but it is masked by the rapid proliferation of opiate receptors, which simultaneously enhance the antinociceptive potency of morphine [16]. The dose–response curve for morphine-induced antinociception in 9-day-old rat pups pretreated with morphine (20 mg/kg) over 4 days is shifted to the right, showing that repeated morphine administration can produce tolerance within the first 2 weeks after birth.

Likewise, it has been shown that 2-week-old rats develop tolerance to continuous subcutaneous morphine infusion within 72 hours [17]. Other studies have shown that opioid tolerance develops within 8–10 days in young adult rats [18,19]. However, there is some debate as to how early a neonatal rat can develop opioid tolerance (e.g. 9 versus 15 days after birth) [16,20].

Results from a study by Laferrière et al [21] indicated that postnatal development did not affect the potency of fentanyl in 6- to 9-day-old rats. The fentanyl pump–implanted animals were observed to develop tolerance to fentanyl, and this tolerance was not affected by gender, developmental changes, fentanyl distribution, or changes in fentanyl metabolism. These results indicate that continuous administration of fentanyl *via* an osmotic minipump can render normal neonatal rats tolerant to and physically dependent on fentanyl within 72 hours [22].

Recently, Zissen et al [23] examined the development of opioid tolerance by intermittent injection or continuous infusion of morphine in postnatal 5- to 8- and 19- to 21-day-old rats and found that different dosages and delivery schedules affected morphine tolerance in an age-dependent manner. These findings suggest that the dose and frequency of opioid administration interact with age in determining the development of tolerance.

### Age-dependent opioid effects in cells

Age-dependent opioid effects have also been observed in cultured neuronal cells. A recent study showed that dorsal root ganglion (DRG) neurons cultured from 10-month-old rats were more sensitive to long-term morphine treatment than neurons from 3-month-old rats; in the neurons from the older rats, lower doses of morphine (10 times lower) and a shorter treatment period (33% shorter) were sufficient to induce significant increases in the immunoreactivity of calcitonin gene-related peptide and substance P [24]. Although the relationship between this age-dependent sensitivity to chronic morphine in cultured DRG neurons and the rate of morphine tolerance development in whole animals is unknown, the impact of aging on the effects of opioids is apparent and significant.

## Molecular and cellular mechanisms of the age-dependency of opioid analgesia and tolerance

**Figure 1:**
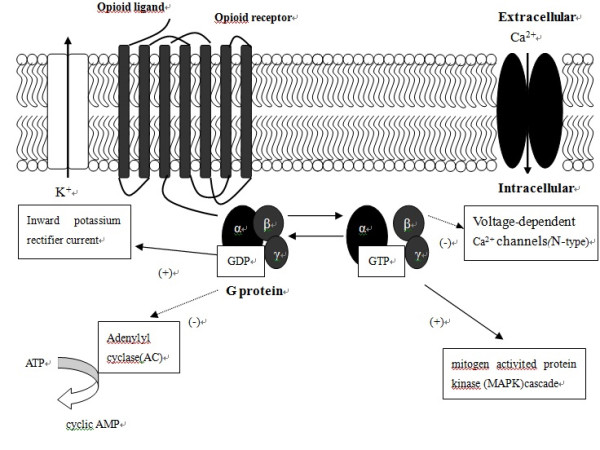
**Opioid receptor signaling pathway.** All four types of opioid receptor are coupled to the inhibitory G protein G_i_ or G_o_, which in turn is regulated by RGS proteins. The analgesic effect of opioid agonists is attributed to the signal transduction through the G protein–mediated second messenger system initiated by the binding of an agonist to an opioid receptor. Once an opioid agonist binds to its specific receptor, the conformation of the opioid receptor changes, and the coupled G_i/o_ protein is subsequently activated. The G_α_ subunit switches from a GDP-bound inactive state to a GTP-bound active state and dissociates from the G_ßγ_ subunits. Activated G subunits then interact with downstream effectors, which further amplify the signal initiated by the opioid agonist and opioid receptor. Those downstream actions include the inhibition of adenylyl cyclase (AC) to reduce the production of cyclic AMP (cAMP), the opening of potassium channels, the inhibition of calcium channels, and the activation of mitogen-activated protein kinase (MAPK) and other kinases.

Mechanisms of opioid tolerance at the molecular and cellular levels are complex. Many of them require a modification of the expression and functions of signaling molecules [25-27]. To explore the molecular and cellular bases of the age-dependency of opioid analgesia and tolerance, we must first have a comprehensive understanding of opioid receptor signaling systems (Figure 1), as well as up-to-date knowledge of the mechanisms of opioid tolerance at the molecular and cellular levels.

Having this knowledge enables us to address two important questions. First, what role does age play in the mechanisms of opioid analgesia and tolerance? Second, how do the development, growth, and aging processes affect the molecular and cellular events described in the previous section?

**Figure 2:**
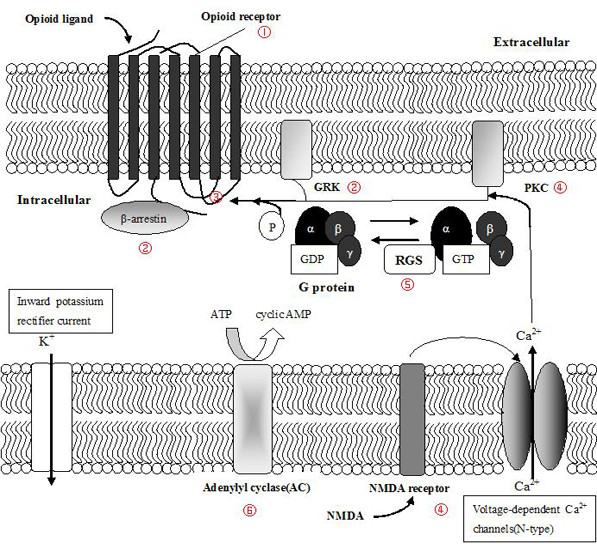
**Molecular and cellular mechanisms of the age-dependency of opioid analgesia and tolerance.** Mechanisms of opioid analgesia and tolerance at the molecular and cellular levels are complex, and many of them require a modification of the expression and functions of signaling molecules. Aging has a significant impact on almost every aspect of the opioid receptor signaling systems that underlie opioid analgesia and tolerance. ① Endogenous opioid peptides and opioid receptors are differentially expressed in different developmental stages, and aging is associated with changes in the number and/or affinity of opioid receptors and opioid receptor-like 1 (ORL1). ② The expression of β-arrestin, which plays a prominent part in opioid receptor desensitization, is determined by neural differentiation and aging. The increased expression of β-arrestin is accompanied by a parallel increase in G protein-coupled receptor kinase (GRK) expression during prenatal development. ③ The phosphorylation of opioid receptors by GRK and the binding of β-arrestin initiate the internalization of the ligand-bound receptors. The internalization of epidermal growth factor (EGF) receptors and interleukin 2 (IL2) receptors and clathrin-associated endocytosis are age-dependent, which implies that the same might be also true for opioid receptor systems. ④ Aging affects the expression and function of the N-methyl-D-aspartic acid (NMDA) receptor and its subunits—calmodulin (CaM) and protein kinase C (PKC) and its various isoforms—as well as other neuropeptides known to have anti-opioid effects. ⑤ The expression, regulation, and function of specific G protein signaling (RGS) members are affected by age during embryonic development and neuronal differentiation. ⑥ Development and aging differentially regulate G protein-mediated adenylate cyclase (AC) signaling. The activities of AC, guanylate cyclase (GC), cyclic AMP (cAMP), phosphodiesterase, and cyclic GMP (cGMP) phosphodiesterase in the frontal cortex and cerebellum show age-related changes.

In response to the first question, evidence shows that development and aging have a significant impact on almost every aspect of the molecular and cellular mechanisms underlying opioid analgesia and tolerance (Figure 2). Some of these effects are described below.

## Opioid receptor signal transduction pathways

Decades of opioid research have led to the magnificent discovery of endogenous opioid peptides and multiple opioid receptors, which play the primary and essential roles in opioid action. In humans and other mammals, there exist at least four families of endogenous opioid peptides. The members of each family are formed from one of four large precursor proteins: prepro-opiomelanocortin, prepro-enkephalin, preprodynorphin, and prepro-nociceptin. Through processes of cleavage by specific peptidases and post-translational modification, a dozen active opioid peptides are derived from the precursors (Table 1). These endogenous opioid peptides play important roles in mediating and modulating the analgesic effect of and tolerance to opioids administered exogenously.

**Table 1:** Endogenous opioid peptides and their precursors

**Opioid Peptide**	**Precursor**
β-Endorphin	Pro-opiomelanocortin
Met-enkephalin	Pro-enkephalin
Leu-enkephalin	Pro-enkephalin
Octapeptide	Pro-enkephalin
Heptapeptide	Pro-enkephalin
Dynorphin 1-8	Pro-dynorphin
Dynorphin 1-17	Pro-dynorphin
α-Neoendorphin	Pro-dynorphin
β-Neoendorphin	Pro-dynorphin
Nociceptin	Prepro-nociceptin
Bocistatin	Prepro-nociceptin

Pharmacological, biochemical, and molecular cloning studies have revealed that there are four different types of opioid receptors: the δ, μ, κ, and opioid receptor-like 1 (ORL1) receptors. They all belong to the G protein–coupled, seven-transmembrane receptor superfamily (GPCR) and share significant sequence homology (more than 60% identical at the amino-acid level) [28]. The δ, μ, and κ types are considered the classical opioid receptors because they are the selective binding sites for commonly-used opioid drugs and mediate typical opioid effects that can be reversed by the specific “pure” opioid antagonist naloxone. In contrast, the ORL1 receptor mediates atypical dual (opioid and anti-opioid) effects that cannot be reversed by naloxone. Thus, the ORL1 receptor can be seen as a divergent and atypical member of the opioid receptor family. The selectivity of the four types of opioid receptors for different endogenous opioid peptides and exogenous opioid drugs is summarized in Table 2.

**Table 2:** Selectivity of Opioid receptors to the Endogenous Opioid Peptides and Exogenous Opioid Drugs

**Opioid receptor**	**Endogenous opioid peptides**	**Opioid drugs**
Μ-receptor	β-endorphin	morphine
	enkephalin	fentanyl
		sufentanil
		D-Ala2,MePhe4,Gly-ol5
		(DAMGO)
δ-receptor	Met-enkephalin	deltorphin
	Leu-enkephalin	[D-Pen2,D-Pen5]enkephalin
		(DPDPE)
κ-receptor	dynorphin A	ethylketocyclazocine (EKC)
	dynorphin B	buprenorphine
		pentazocine
		U 50,488
ORL1-receptor	nociceptin	None

Opioid receptors of all four types are coupled to the inhibitory G protein G_i_ or Go, which is in turn regulated by RGS proteins. The analgesic effect of opioid agonists is attributed to signal transduction through the G protein-mediated second messenger system initiated by the binding of agonist to opioid receptor. Once an opioid agonist binds to its specific receptor, the conformation of the opioid receptor changes, and the coupled G_i/o_ protein is subsequently activated. The G_α_ subunit switches from a GDP-bound inactive state to a GTP-bound active state and dissociates from the G_ßγ_ subunits. Activated G subunits then interact with downstream effectors, which further amplify the signal initiated by the opioid agonist and opioid receptor. Those downstream actions include the inhibition of adenylyl cyclase (AC) to reduce the production of cyclic AMP (cAMP), the opening of potassium channels, the inhibition of calcium channels, and the activation of mitogen-activated protein kinase (MAPK) and other kinases.

The agonist-bound opioid receptor goes through internalization for signaling and recycling itself. Opioid receptor internalization is assisted by RGS proteins [29]. The termination of opioid signaling results from the hydrolysis of GTP by a GTPase; this process is accelerated by specific RGS proteins, which return the G_α_ subunit to its GDP-bound inactive form. The processes of internalization, recycling, and inactivation resets opioid receptors and G proteins, readying them to transduce the next signal when another opioid agonist binds to the receptor.

Evidence shows that all four types of opioid receptor are involved in opioid analgesia and tolerance. Each of the four types of opioid agonist produces analgesia (or hyperalgesia in the case of nociceptin) and tolerance by binding to its respective receptor. In addition, the multiple types of opioid receptors interact with each other to enhance or attenuate opioid analgesia and tolerance. For example, studies have shown that morphine analgesia is greatly enhanced by activation of δ or κ opioid receptors [30] and that the ORL1 agonist nociceptin/orphanin FQ attenuates morphine analgesia and accelerates morphine tolerance [31].

### Age-dependent expression and function of endogenous opioid peptides and multiple opioid receptors

It has been well documented that the expression and distribution of the endogenous opioid peptides—prepro-enkephalin, prepro-endorphin, preprodynorphin, and prepronociceptin—are age-related [32,33]. It has also been shown that opioid receptors and ORL1 are differentially expressed in different developmental stages and ages [34,35]. During the postnatal preweaning period, there is a progressive increase in the number of μ receptors in the whole brain, and this increase is correlated with an increase in the antinociceptive efficacy of morphine (14/4). Studies by Rahman et al [36]. showed that the numbers of μ, δ, and κ opioid receptor binding sites in the spinal cord increase from 0 postnatal days, reach a peak at 7 postnatal days, and then progressively decrease, reaching adult levels at 56 postnatal days. Zhang and Pasternak [37] reported that high-affinity κ opioid receptor binding sites in the spinal cord increase three-fold from 2 postnatal days to 14 postnatal days; the majority of this increase occurs on or around 5 postnatal days. These findings indicate that there are parallel changes in the numbers and/or affinity of opioid receptors and the strength of opioid-induced analgesia over the first few postnatal weeks of life. Another group found that aging is associated with an increased affinity (decreased dissociation constant Kd) of the μ opioid receptors without a significant effect on the number of μ receptors [38].

However, controversy exists, as the study from Bardo et al [39] shows that an alteration in the opiate system during development does not necessarily produce a concomitant alteration in the behavioral efficacy of morphine. This conclusion was drawn from the finding that the long-term administration of morphine did not alter the ligand binding of opioid receptors in certain areas of the brain in rats 1 to 21 days of age.

A large body of evidence has shown that development and age have a significant impact on the expression and functional activities of opioid receptors and ORL1. Table 3 summarizes the major findings.

**Table 3:** Classification of RGS proteins

**Subfamily**	**Members**	**Common features outside of RGS domain**
RZ (or A) string	GAIP/RGS19, RGSZ1/RGS20, RGSZ2/RGS17, Ret-RGS1	N-terminal cysteine
R4 (or B)	RGS1, RGS2, RGS3, RGS4, RGS5, RGS8, RGS13, RGS16, RGS18	without specified domains or just an N-terminal amphipathic helix
R7 (or C)	RGS6, RGS7, RGS9, RGS11	a GGL (G-like) domain and a DEP domain
R12 (or D)	RGS10, RGS12, RGS14	may contain PDZ, PTB, or RBD domains
RA (or E)	axin, conductin	GSK binding, ß-catenin binding, PP2A homology, and dimerization domains
GEF (or F)	p115-RhoGEF, PDZ-RhoGEF, LARG	DH and PH domains
GRK (or G)	GRK1, GRK2, GRK3, GRK4, GRK5, GRK6, GRK7	G-protein receptor kinase domain and PH domain

### Effect of age on opioid receptor phosphorylation and desensitization in the development of opioid tolerance

The binding of an agonist to an opioid receptor induces two events: the activation of the opioid signal transduction pathway and the modulation (including but not limited to phosphorylation and desensitization) of the opioid receptor itself. Opioid receptor phosphorylation and desensitization have been linked to the development of opioid tolerance [40]. Continued exposure to an agonist leads to the phosphorylation of opioid receptors by G protein–coupled receptor kinases (GRKs). The phosphorylated receptor is then bound by β-arrestin, a member of the arrestin family that can recognize both GRK phosphorylation sites on the receptor and the activated conformation of the receptor. The phosphorylation of the opioid receptor and the binding of β-arrestin result in the uncoupling of the opioid receptor from G proteins, which leads to a desensitization of the opioid receptor and a reduction of opioid agonist’s efficacy [41].

Among the members of the arrestin family, β-arrestin 1 (i.e. arrestin 2) and β-arrestin 2 (i.e. arrestin 3) contribute to the regulation of the majority of GPCRs. Long-term morphine treatment of cells that express μ-opioid receptor (MOR) leads to the attenuation of β-arrestin 1 and β-arrestin 2, which subsequently desensitize the activated receptors and facilitate the internalization of inactivated receptors and the recycling of resensitized receptors back to the cell surface [42]. Our understanding of these effects, along with some other research findings, strongly suggest that GRK and arrestin play essential roles in the processes underlying MOR desensitization, which may contribute to the development of opioid tolerance.

The immunodensities of GRK2, GRK6, and β-arrestin 2 in the prefrontal cortex were found to be significantly lower in opiate addicts than in controls [43]. This finding indicates that opioid tolerance is associated with a down-regulation of brain MOR and a regulation of GRK 2/6 and β-arrestin 2.

It is generally believed that the efficiency of GPCR signaling correlates with the concentration of receptors, G-proteins, and effectors, whereas the rate of receptor desensitization correlates with the concentration of relevant GRKs and arrestins. GRK2 and GRK6 play important roles in the phosphorylation of G proteins and in the regulation of opioid receptors. In human prefrontal cortex, the immunodensities of GRK2/6 and β-arrestin 2 appear to decline significantly with aging (i.e. between the ages of 16–87 y) [44].

The expression of arrestin 2, which plays a prominent part in opioid receptor desensitization, is determined by neural differentiation and aging. A study performed in rat embryos detected a steady increase in arrestin 2 expression during prenatal development. At early stages of prenatal development, the concentrations of the two arrestin isoforms are similar. The increase in arrestin 2 is accompanied by a parallel increase in GRK5 expression, whereas the expression of other GRK subtypes changes very little [45].

### Effects of age on opioid receptor internalization in opioid tolerance

It is believed that the number of functional opioid receptors on the cell membrane surface determines the magnitude of an opioid’s effects, including analgesia. An orthodox hypothesis is that binding with an agonist induces opioid receptor internalization and downregulation, which reduce the number of available opioid receptors on the cell surface and, therefore, reduce the effect of the opioid agonist and facilitate the development of tolerance. This notion has been supported by plentiful data on opioid tolerance [46-48].

However, some recent studies have offered a revised conception of the relationship between opioid receptor internalization and opioid tolerance. This new model suggests that the opioid receptor internalization actually prevents or delays the development of opioid tolerance, and that the effectiveness with which opioid agonists induce μ opioid–receptor internalization is inversely proportional to their potency to induce tolerance. *In vitro* data show that morphine is inefficient in inducing μ opioid–receptor internalization but is potent in producing tolerance. In contrast, the selective μ agonist DAMGO induces μ receptor internalization efficiently and does not produce tolerance readily [49].

The phosphorylation of opioid receptors by GRK and the binding of β-arrestin initiate the internalization of the ligand-bound receptors and a subsequent recycling of the receptors back to the cell surface (80% of internalized receptors are recycled from endosomes to the plasma membrane by dephosphorylation). These findings strongly support the idea that receptor internalization reduces tolerance *in vivo* by facilitating the recycling and resensitization of receptors.

However, this model is still in a very early stage of development, and there are several facts that appear to contradict it. For one, the timings of *in vitro* opioid receptor internalization and *in vivo* opioid tolerance development are not correlated at all. Furthermore, it has become clear that the potency of a given opioid agonist to induce μ opioid–receptor internalization is not a fixed property; instead, it is tissue-specific and cell-type–dependent. Recent studies show that morphine, the most inefficient inducer of μ opioid receptor internalization in *in vitro* cell expression systems, can actually induce the rapid internalization of a significant portion of μ opioid receptors in striatum neuronal cells [50] and in mouse periaqueductal grey matter (PAG) neurons [51].

Opioid receptor internalization is one of the most important events in opioid tolerance. Although there is no direct evidence that the internalization of opioid receptors is affected by age, some studies have shown that the ligand-induced internalization of epidermal growth factor (EGF) and interleukin 2 (IL2) receptors and clathrin-associated endocytosis are age-dependent [52-54]. From this evidence, one could infer that internalization might also occur in opioid receptor systems.

### Effect of age on the expression and function of NMDA receptor and other proteins that play important roles in opioid tolerance

It has been demonstrated that MOR and NMDA are colocalized in individual neurons in many areas of the central nervous system [55-57]. The interaction involves intracellular second messengers that mediate opioid action, leading to analgesia and the development of tolerance. MOR activation initiates multiple cellular signaling cascades that result in protein kinase C (PKC) γ-subtype translocation [58] and the inhibition of Ca^2+^ channels [59]. In contrast, NMDA receptor activation is associated with PKC activation and an increase in intracellular Ca^2 +^[60]. NMDA receptors attenuate opioid receptor function by facilitating Ca^2+^ entry and PKC phosphorylation of the G_iα2_ protein, resulting in opioid receptor–G protein uncoupling [61].

Uncoupling of the MOR–G protein, rather than receptor internalization, has been implicated as a mechanism for tolerance to morphine [62]. Inhibition of NMDA receptors, which prevents or reduces MOR–G protein uncoupling, may enhance opioid analgesia and delay the development of tolerance [63,64].

In addition to NMDA receptors, other anti-opioid systems have been discovered [65]. Several neuropeptides, including cholecystokinin (CCK), neuropeptide FF (NPFF), and nociceptin (orphanin FQ), have a pharmacological effect that negatively modulates the opioid system. Prolactin-releasing peptide (PrRP) has recently been identified as the natural agonist of GPR10, which was previously considered an orphan receptor. This study identified the PrRP-GPR10 system as a potent negative modulator of the opioid system, so the PrRP-GPR10 system may be involved in the development of opioid tolerance and dependence.

Neurokinins and calcitonin gene-related peptide (CGRP) are expressed in primary sensory afferents and have thus been proposed to play important roles in nociceptive sensation. Menard et al [66] investigated the expression of CGRP and its receptors in the dorsal horn of the spinal cord during the development of tolerance to continuous intrathecal administration of morphine. In animals that developed opioid tolerance, there was a significant increase in CGRP-like immunostaining and a decrease (30-45%) in [125I]human CGRP α binding in the laminae I, II, and III of the dorsal horn of the spinal cord. These changes suggest that CGRP may play a role in the development of opioid tolerance.

In addition, Powell et al [67] discovered that in rats that were given repeated doses of morphine, coadministration of SR140333—a selective substance P receptor (neurokinin-1) antagonist—augmented the acute effects of morphine, prevented morphine tolerance, and reversed established tolerance. These findings suggest that the activity of neurokinin also contributes to the induction of opioid analgesic tolerance.

Development and aging differentially regulate the expression and function of the NMDA receptor and its subunits [44]. A study showed that NMDA antagonist was not effective in blocking the development of morphine tolerance in 7-day-old rats, was partially effective in 14-day-old rats, and was fully effective in 21-day-old or older rats [68]. These data suggest that there is a transition age, around the second postnatal week in the rat, at which NMDA receptors begin to play a role in the development of morphine tolerance. One possible explanation for opioid tolerance in newborn rats is that other mechanisms, such as NO production, activate the intracellular Ca^2+^ release and evoke the Ca^2+^-dependent second messenger system. A second possibility is that channels besides the NMDA receptors allow significant Ca^2+^ production in the infant CNS, thereby facilitating opiate action.

The morphine tolerance observed in newborn rats may be mediated by metabotropic glutamate receptors (mGluRs), because mGluRs are coupled to various second messengers, including Ca^2+^ cascades. The AMPA receptor may also activate the Ca^2+^-dependent second messenger systems in neural circuits involved in opiate tolerance in newborn rats. A study showed that treatment with the selective AMPA receptor antagonist NBQX or the group II mGluR agonist DCG-IV effectively suppressed the expression of morphine-induced tolerance and dependence in infant rats. These effects were not age-dependent [68].

Spinal glutamate, nitric oxide, cyclooxygenase (COX), and prostaglandin-related systems are all known to be activated by opioid-related analgesia. The enzymatic activity of COX, and to a lesser extent nitric oxide, also contributes to the development of spinal morphine tolerance. Other neuropeptides known to have anti-opioid effects (CGRP, substance P [SP], neuropeptide Y [NPY], galanin) have also been shown to have age-dependent expression and activities. One study showed that CGRP-like immunoreactivity was significantly increased in the primary afferents of the spinal dorsal horn during the development of morphine tolerance [24]. In addition, DRG neurons cultured from 10-month-old rats were more sensitive to morphine treatment, in that lower concentrations and shorter treatment periods could induce apparent increases in the number of CGRP-and SP-IR neurons, suggesting that aging plays a role in the responsiveness of DRG neurons to repeated morphine exposure. The greater sensitivity of morphine-mediated CGRP and SP induction in cultured DRG neurons from older rats suggests that morphine tolerance may be more likely to develop in the elderly.

Calmodulin (CaM) plays an important role in opioid receptor signaling. Age-induced changes in the CaM system were observed by Hoskins et al [69]. These observations included the following differences: First, CaM levels were lowest in young rats, higher in old rats, and highest in mature rats. Second, Ca^2+^-Mg^2+^ ATPase activity was progressively higher in young, mature, and old rats. Third, particulate protein kinase activity was progressively lower in young, mature, and old rats.

Development and aging differentially regulate the expression and function of PKC and its various isoforms, as well as other protein kinases [70]. Taken together, these findings make it plausible to hypothesize that age-dependent changes in the expression and function of the major factors are the molecular and cellular bases of age-dependent opioid analgesia and tolerance.

### Age-related differential expression and function of G proteins and RGS proteins in opioid tolerance

RGS proteins are a family of cellular proteins that contain a homologous RGS domain of approximately 120 amino acids in length. RGS proteins include GTPase-accelerating protein (GAP) activity within their characteristic RGS domain and various other receptor signaling-related properties of their other functional domains. Multiple RGS proteins have been shown to negatively regulate G protein–mediated opioid signaling, facilitate opioid receptor desensitization and internalization, and affect the rate at which opioid tolerance develops [71]. RGS proteins specifically interact with G_α_ subunits and enhance the intrinsic GTPase activity of G_α_ to accelerate GTP hydrolysis, thereby facilitating the switch of G_α_ from a GTP-bound active state to a GDP-bound inactive state. It was not until recently that RGS proteins were recognized as key players in opioid signaling and tolerance. An increasing number of studies show that specific RGS proteins, especially GAIP/RGS19, RGS2, RGS4, RGS8, and RGS9-2, play crucial roles in opioid receptor signaling and opioid tolerance (Table 4) [72]. They not only inactivate G protein, which terminates opioid action, but also function as active components in opioid receptor desensitization, internalization, recycling, and degradation [73].

**Table 4:** Effect of age on the expression and functional properties of multiple opioid receptors

**Opioid receptor type**	**Animal models and experimental means**	**Observed age-dependent expression and activities of opioid receptors**
μ	DAMGO and dihydromorphine binding assays in brains of mice of various ages	Bmax values and selectivity for -selective opioid ligands change as a function of age
	DAMGO binding with light and heavy membranes of rat brain	The subcellular distribution of opioid receptors changes with age
	DAMGO binding in the spinal cord of rats of different ages	The Kd value for DAMGO is significantly higher in the aged rats than in the young and mature rats, indicating a decreased affinity of spinal opioid receptors for DAMGO
	Effect of opioid agonists on warm water–stimulated tail-withdrawal in young (3 months) and old (24 months) male rats	Old male rats are more sensitive to the antinociceptive effects of opioids than young ones; the age-related differences in opioid sensitivity are most apparent when lower-efficacy opioids and higher nociceptive intensities are tested
	EM ICC with rat caudate-putamen nucleus	The developmental expression of opioid receptors parallels asymmetric synapse formation
δ	Quantitative autoradiography with opioid receptor binding in guinea pig brain	With age, opioid receptor density decreases in the globus pallidus and increases in the neocortex
	EM ICC with rat caudate-putamen nucleus	Opioid receptor expression gradually increases from birth to adulthood and correlates with synapse formation
	Agonists DSLET and DPDPE used to stimulate high-affinity GTPase activity in young (4 weeks) and old (16 weeks) guinea pig striatal membranes	Agonists can stimulate high-affinity GTPase activity in striatal membranes from old guinea pigs but not from young ones, indicating age-dependent opioid receptor-G protein functional coupling
κ	Quantitative autoradiography with κ opioid receptor binding in guinea pig brain	Expression of opioid receptors decreases with age
	IP injection of selective κ opioid agonist U50,488 H in young (6–8 weeks) and old (21–22 months) mice	Qualitative sex differences in opioid analgesia in the mice are dependent on age
	Tested effect of opioid agonists on the warm water-stimulated tail-withdrawal in young (3 months) and old (21 months) male rats	Aged male rats are more sensitive than young ones to the antinociceptive effects of opioid agonists
ORL1	In situ hybridization and autoradiography with human, rat, and mouse brains	Differential expression of ORL1 found in developing and adult brains

IP, intraperitoneal

The expression and activities of inhibitory G proteins that are coupled to opioid receptors are also age-dependent [74]. Development and aging differentially regulate G protein-mediated AC signaling; the activities of adenylate cyclase, guanylate cyclase, cyclic AMP phosphodiesterase, and cyclic GMP phosphodiesterase in the frontal cortex and cerebellum show age-related changes during morphine treatment. Such changes are not due to any age-related changes in the pharmacokinetics of morphine [75].

The importance of development and age in determining the expression and function of RGS genes and proteins has just begun to draw attention. Recent studies show that the expression, regulation, and function of specific RGS members are indeed affected by development and age. During embryonic development and neuronal differentiation, the expression of RGS4 occurs in a highly dynamic and transient manner in a small set of peripheral and central neuronal precursor cells, and it is regulated by the neural type-specific transcription factor Phox2b [76]. However, in the developing postnatal brain, RGS4 expression increases in the deep neuronal layers of the neocortex, the CA1/2 area of the hippocampus, and the cerebellum [77]. In the adult brain, RGS4 continues its dense expression in the neocortex, thalamus, and cerebellum, but not in the hippocampus. The expressions of RGS2 and RGS7 are differentially regulated in the embryonic, early postnatal, and adult brain in a region-specific manner [77-79]. Interestingly, the alternative splicing pattern of RGS9 is regulated by development and age. During embryonic and early postnatal development, two RGS9 transcripts of approximately 1.4 kb and 1.8 kb are detected in whole brain. After postnatal day 10, the expression of 1.8-kb transcript increases progressively until adulthood and becomes concentrated in the striatum, while 1.4-kb transcript expression gradually decreases to undetectable levels [80]. Recently, immunochemical staining with specific RGS9 antibody RGS9 proteins (predominantly RGS9-2, as the observed distributions showed) were found to be differentially expressed in the nervous system, notably in the nociceptive system, of young and old rats, which may shed light on the mechanisms of age-dependent opioid analgesia and tolerance [81]. The functions and activities of RGS proteins may also be age-dependent. It is reported that RGS1 can significantly increase GABAergic agonist-stimulated GTPase activity in the cerebral cortex of 90-day-old rats but cannot do so in 12-day-old rats [82]. These findings strongly support the hypothesis that age plays an important part in RGS expression and function.

RGS proteins also play roles in regulating neuronal development, cell proliferation, differentiation, and plasticity [83-86]. In addition, the expression of certain RGS proteins is found to be modulated by some age-related diseases, such as Parkinson's and Alzheimer's diseases [87,88]. Such age-associated changes in RGS protein expression may in turn alter the effects of opioids.

### Age-related differences in opioid pharmacokinetics

One important issue regarding the age-dependence of opioid tolerance and recovery is the difference in opioid drug metabolism (pharmacokinetics) between younger and older individuals. It has been well established that the rates of metabolism, blood–brain transport, and clearance of opioids in different age groups of animals and humans differ significantly [89-91]. These differences substantially affect the *in vivo* pharmacological effects of opioids. However, several studies have demonstrated that the development of tolerance to the analgesic and hyperthermic effects of morphine is not related to its pharmacokinetics in serum but may be related to the modification of opioid receptor signal transduction pathways in the CNS [92,93].

## Conclusions

Age is an important physiological factor that influences opioid drug action. The subject of opioid tolerance and aging has drawn great attention and interest. To understand the molecular mechanisms of the age-dependency of opioid tolerance is important for both basic scientific research and clinical practice. A thorough investigation of the patterns of tolerance induced by various type-selective opioids, as well as the differential expression and functions (including internalization) of multiple opioid receptors during opioid tolerance development, in animals of different ages may lead to new insights into the pharmaceutical application of type-selective opioid drugs for improving opioid analgesia and delaying tolerance occurrence in chronic pain therapy. In addition to providing novel insights into the best opioid agonists to use in various age groups of patients to avoid rapid tolerance development, further defining the concept of age-dependent tolerance will help to educate physicians who treat with chronic pain patients of different ages. Currently, little attention is paid to the age of the patient when clinicians decide whether to prescribe daily opioids to patients with nonmalignant pain conditions. Upfront discussions of the appropriate dosing of opioids in young patients for chronic pain conditions can greatly benefit from published data regarding the age-dependent mechanisms of opioid tolerance. In addition, the apparent age-dependency of opioid analgesia and tolerance suggests that age should be included as a parameter in studies of opioid analgesia and tolerance.

## Abbreviations

RGS: regulators of G protein signaling; NMDA: N-methyl-D-aspartic acid; VAS: visual analog scale; DRG: dorsal root ganglion; ORL1: opioid receptor-like 1; GPCR: G protein-coupled, seven-transmembrane receptor superfamily; AC: adenylyl cyclase; cAMP: cyclic AMP; MAPK: mitogen-activated protein kinase; GRKs: G protein-coupled receptor kinases; MOR: μ-opioid receptor; PAG: periaqueductal grey matter; EGF: epidermal growth factor; IL2: interleukin 2; PKC: protein kinase C; CCK: cholecystokinin; NPFF: neuropeptide FF; PrRP: prolactin-releasing peptide; COX: cyclooxygenase; CGRP: calcitonin gene-related peptide; mGluRs: metabotropic glutamate receptors; NPY: neuropeptide Y; SP: substance P; GAP: GTPase-accelerating protein.

## Competing interests

None of the authors has any financial or scientific conflicts of interest with regard to the research described in this manuscript.

## Authors’ contributions

ZJ conceived of the review and drafted the manuscript. XX participated in the design of the figures and helped to draft the manuscript. XGX and PP participated in the design of the review and helped to revise the manuscript. HYG helped to revise the manuscript. All authors have read and approved the final manuscript.

## References

[B1] Vigano A Bruera E Suarez-Almazor ME Age, pain intensity, and opioid dose in patients with advanced cancer Cancer 1998 83 1244 1250 10.1002/(SICI)1097-0142(19980915)83:6<1244::AID-CNCR26>3.0.CO;2-4 9740092

[B2] Hall S Gallagher RM Gracely E Knowlton C Wescules D The terminal cancer patient: effects of age, gender, and primary tumor site on opioid dose Pain Med 2003 4 125 134 10.1046/j.1526-4637.2003.03020.x 12873262

[B3] Clark JD Chronic pain prevalence and analgesic prescribing in a general medical population J Pain Symptom Manage 2002 23 131 137 10.1016/S0885-3924(01)00396-7 11844633

[B4] Savage SR Opioid therapy of chronic pain: assessment of consequences Acta Anaesthesiol Scand 1999 43 909 917 10.1034/j.1399-6576.1999.430908.x 10522738

[B5] Paice JA Penn RD Shott S Intraspinal morphine for chronic pain: a retrospective, multicenter study J Pain Symptom Manage 1996 11 71 80 10.1016/0885-3924(95)00099-2 8907137

[B6] Mystakidou K Parpa E Tsilika E Mavromati A Smyrniotis V Georgaki S Vlahos L Long-term management of noncancer pain with transdermal therapeutic system-fentanyl J Pain 2003 4 298 306 14622686 10.1016/s1526-5900(03)00632-1

[B7] Robinson RC Gatchel RJ Polatin P Deschner M Noe C Gajraj N Screening for problematic prescription opioid use Clin J Pain 2001 17 220 228 10.1097/00002508-200109000-00006 11587112

[B8] Buntin-Mushock C Phillip L Moriyama K Palmer PP Age-dependent opioid escalation in chronic pain patients Anesth Analg 2005 100 1740 1745 10.1213/01.ANE.0000152191.29311.9B 15920207

[B9] Moulin DE Iezzi A Amireh R Sharpe WK Boyd D Merskey H Randomised trial of oral morphine for chronic non-cancer pain Lancet 1996 347 143 147 10.1016/S0140-6736(96)90339-6 8544547

[B10] Jourdan D Pickering G Marchand F Gaulier JM Alliot J Eschalier A Impact of ageing on the antinociceptive effect of reference analgesics in the Lou/c rat Br J Pharmacol 2002 137 813 820 10.1038/sj.bjp.0704944 12411412 PMC1573564

[B11] O'Callaghan JP Holtzman SG Prenatal administration of morphine to the rat: tolerance to the analgesic effect of morphine in the offspring J Pharmacol Exp Ther 1976 197 533 544 932990

[B12] Windh RT Little PJ Kuhn CM The ontogeny of mu opiate tolerance and dependence in the rat: antinociceptive and biochemical studies J Pharmacol Exp Ther 1995 273 1361 1374 7791109

[B13] Zhu H Barr GA Ontogeny of NMDA receptor-mediated morphine tolerance in the postnatal rat Pain 2003 104 437 447 10.1016/S0304-3959(03)00051-4 12927616

[B14] Nozaki M Akera T Lee CY Brody TM The effects of age on the development of tolerance to and physical dependence on morphine in rats J Pharmacol Exp Ther 1975 192 506 512 1168253

[B15] Wang Y Mitchell J Moriyama K Kim KJ Sharma M Xie GX Palmer PP Age-dependent morphine tolerance development in the rat Anesth Analg 2005 100 1733 1739 10.1213/01.ANE.0000152192.23851.40 15920206

[B16] Van Praag H Frenk H Evidence for opiate tolerance in newborn rats Brain Res Dev Brain Res 1991 60 99 102 10.1016/0165-3806(91)90160-k 1914149

[B17] Thornton SR Wang AF Smith FL Characterization of neonatal rat morphine tolerance and dependence Eur J Pharmacol 1997 340 161 167 10.1016/S0014-2999(97)01434-9 9537810

[B18] Trujillo KA Akil H Inhibition of morphine tolerance and dependence by the NMDA receptor antagonist MK-801 Science 1991 251 85 87 10.1126/science.1824728 1824728

[B19] Fan GH Wang LZ Qiu HC Ma L Pei G Inhibition of calcium/calmodulin-dependent protein kinase II in rat hippocampus attenuates morphine tolerance and dependence Mol Pharmacol 1999 56 39 45 10385682 10.1124/mol.56.1.39

[B20] Fanselow MS Cramer CP The ontogeny of opiate tolerance and withdrawal in infant rats Pharmacol Biochem Behav 1988 31 431 438 10.1016/0091-3057(88)90370-X 3244719

[B21] Laferriere A Colin-Durand J Moss IR Ontogeny of respiratory sensitivity and tolerance to the mu-opioid agonist fentanyl in rat Brain Res Dev Brain Res 2005 156 210 217 10.1016/j.devbrainres.2005.03.002 16099308

[B22] Thornton SR Smith FL Characterization of neonatal rat fentanyl tolerance and dependence J Pharmacol Exp Ther 1997 281 514 521 9103539

[B23] Zissen MH Zhang G McKelvy A Propst JT Kendig JJ Sweitzer SM Tolerance, opioid-induced allodynia and withdrawal associated allodynia in infant and young rats Neuroscience 2007 144 247 262 10.1016/j.neuroscience.2006.08.078 17055659 PMC1858640

[B24] Ma W Zheng WH Kar S Quirion R Morphine treatment induced calcitonin gene-related peptide and substance P increases in cultured dorsal root ganglion neurons Neuroscience 2000 99 529 539 10.1016/S0306-4522(00)00226-8 11029544

[B25] Przewlocki R Opioid abuse and brain gene expression Eur J Pharmacol 2004 500 331 349 10.1016/j.ejphar.2004.07.036 15464044

[B26] Ammon-Treiber S Hollt V Morphine-induced changes of gene expression in the brain Addict Biol 2005 10 81 89 10.1080/13556210412331308994 15849022

[B27] Bagley EE Chieng BC Christie MJ Connor M Opioid tolerance in periaqueductal gray neurons isolated from mice chronically treated with morphine Br J Pharmacol 2005 146 68 76 10.1038/sj.bjp.0706315 15980868 PMC1576256

[B28] Przewlocki R Przewlocka B Opioids in neuropathic pain Curr Pharm Des 2005 11 3013 3025 10.2174/1381612054865055 16178760

[B29] Hepler JR Emerging roles for RGS proteins in cell signalling Trends Pharmacol Sci 1999 20 376 382 10.1016/S0165-6147(99)01369-3 10462761

[B30] Wang D Sun X Bohn LM Sadee W Opioid receptor homo- and heterodimerization in living cells by quantitative bioluminescence resonance energy transfer Mol Pharmacol 2005 67 2173 2184 10.1124/mol.104.010272 15778451

[B31] Lutfy K Hossain SM Khaliq I Maidment NT Orphanin FQ/nociceptin attenuates the development of morphine tolerance in rats Br J Pharmacol 2001 134 529 534 10.1038/sj.bjp.0704279 11588106 PMC1572978

[B32] De Vries TJ Jonker AJ Voorn P Mulder AH Schoffelmeer AN Adaptive changes in rat striatal preproenkephalin expression and dopamine-opioid interactions upon chronic haloperidol treatment during different developmental stages Brain Res Dev Brain Res 1994 78 175 181 10.1016/0165-3806(94)90024-8 7913003

[B33] Tseng LF Collins KA Wang Q Differential ontogenesis of thermal and mechanical antinociception induced by morphine and beta-endorphin Eur J Pharmacol 1995 277 71 76 10.1016/0014-2999(95)00064-R 7635176

[B34] Volterra A Brunello N Restani P Galli CL Racagni G Ontogenetic studies on mu, delta and kappa opioid receptors in rat brain Pharmacol Res Commun 1986 18 979 990 10.1016/0031-6989(86)90100-1 3027723

[B35] Hoskins DL Gordon TL Crisp T The effects of aging on mu and delta opioid receptors in the spinal cord of Fischer-344 rats Brain Res 1998 791 299 302 10.1016/S0006-8993(98)00034-1 9593954

[B36] Rahman W Dashwood MR Fitzgerald M Aynsley-Green A Dickenson AH Postnatal development of multiple opioid receptors in the spinal cord and development of spinal morphine analgesia Brain Res Dev Brain Res 1998 108 239 254 10.1016/s0165-3806(98)00054-6 9693800

[B37] Zhang AZ Pasternak GW Ontogeny of opioid pharmacology and receptors: high and low affinity site differences Eur J Pharmacol 1981 73 29 40 10.1016/0014-2999(81)90142-4 6274643

[B38] Hoskins B Ho IK Age-induced differentiation of morphine's effect on cyclic nucleotide metabolism Neurobiol Aging 1987 8 473 476 10.1016/0197-4580(87)90043-1 2891056

[B39] Bardo MT Bhatnagar RK Gebhart GF Differential effects of chronic morphine and naloxone on opiate receptors, monoamines, and morphine-induced behaviors in preweanling rats Brain Res 1982 256 139 147 6286051 10.1016/0165-3806(82)90037-2

[B40] Pitcher JA Freedman NJ Lefkowitz RJ G protein-coupled receptor kinases Annu Rev Biochem 1998 67 653 692 10.1146/annurev.biochem.67.1.653 9759500

[B41] Ferguson SS Barak LS Zhang J Caron MG G-protein-coupled receptor regulation: role of G-protein-coupled receptor kinases and arrestins Can J Physiol Pharmacol 1996 74 1095 1110 10.1139/y96-124 9022829

[B42] Luttrell LM Lefkowitz RJ The role of beta-arrestins in the termination and transduction of G-protein-coupled receptor signals J Cell Sci 2002 115 455 465 11861753 10.1242/jcs.115.3.455

[B43] Ferrer-Alcon M La Harpe R Garcia-Sevilla JA Decreased immunodensities of micro-opioid receptors, receptor kinases GRK 2/6 and beta-arrestin-2 in postmortem brains of opiate addicts Brain Res Mol Brain Res 2004 121 114 122 14969742 10.1016/j.molbrainres.2003.11.009

[B44] Ontl T Xing Y Bai L Kennedy E Nelson S Wakeman M Magnusson K Development and aging of N-methyl-D-aspartate receptor expression in the prefrontal/frontal cortex of mice Neuroscience 2004 123 467 479 10.1016/j.neuroscience.2003.09.006 14698754

[B45] Gurevich EV Benovic JL Gurevich VV Arrestin2 expression selectively increases during neural differentiation J Neurochem 2004 91 1404 1416 10.1111/j.1471-4159.2004.02830.x 15584917

[B46] Bhargava HN Gulati A Down-regulation of brain and spinal cord mu-opiate receptors in morphine tolerant-dependent rats Eur J Pharmacol 1990 190 305 311 10.1016/0014-2999(90)94194-3 2176984

[B47] Bernstein MA Welch SP mu-Opioid receptor down-regulation and cAMP-dependent protein kinase phosphorylation in a mouse model of chronic morphine tolerance Brain Res Mol Brain Res 1998 55 237 242 9582426 10.1016/s0169-328x(98)00005-9

[B48] Stafford K Gomes AB Shen J Yoburn BC mu-Opioid receptor downregulation contributes to opioid tolerance in vivo Pharmacol Biochem Behav 2001 69 233 237 10.1016/S0091-3057(01)00525-1 11420091

[B49] Koch T Widera A Bartzsch K Schulz S Brandenburg LO Wundrack N Beyer A Grecksch G Hollt V Receptor endocytosis counteracts the development of opioid tolerance Mol Pharmacol 2005 67 280 287 10.1124/mol.104.004994 15475572

[B50] Haberstock-Debic H Kim KA Yu YJ von Zastrow M Morphine promotes rapid, arrestin-dependent endocytosis of mu-opioid receptors in striatal neurons J Neurosci 2005 25 7847 7857 10.1523/JNEUROSCI.5045-04.2005 16120787 PMC6725258

[B51] Rodriguez-Munoz M de la Torre-Madrid E Gaitan G Sanchez-Blazquez P Garzon J RGS14 prevents morphine from internalizing Mu-opioid receptors in periaqueductal gray neurons Cell Signal 2007 19 2558 2571 10.1016/j.cellsig.2007.08.003 17825524

[B52] Hara H Tanaka T Negoro S Deguchi Y Nishio S Saiki O Kishimoto S Age-related changes of expression of IL-2 receptor subunits and kinetics of IL-2 internalization in T cells after mitogenic stimulation Mech Ageing Dev 1988 45 167 175 10.1016/0047-6374(88)90106-6 3264597

[B53] Liu L Turner JR Yu Y Khan AJ Jaszewski R Fligiel SE Majumdar AP Differential expression of EGFR during early reparative phase of the gastric mucosa between young and aged rats Am J Physiol 1998 275 G943 950 9815022 10.1152/ajpgi.1998.275.5.G943

[B54] Blanpied TA Scott DB Ehlers MD Age-related regulation of dendritic endocytosis associated with altered clathrin dynamics Neurobiol Aging 2003 24 1095 1104 10.1016/j.neurobiolaging.2003.04.004 14643381

[B55] Gracy KN Svingos AL Pickel VM Dual ultrastructural localization of mu-opioid receptors and NMDA-type glutamate receptors in the shell of the rat nucleus accumbens J Neurosci 1997 17 4839 4848 9169542 10.1523/JNEUROSCI.17-12-04839.1997 PMC6573336

[B56] Wang H Gracy KN Pickel VM Mu-opioid and NMDA-type glutamate receptors are often colocalized in spiny neurons within patches of the caudate-putamen nucleus J Comp Neurol 1999 412 132 146 10.1002/(SICI)1096-9861(19990913)412:1<132::AID-CNE10>3.0.CO;2-B 10440715

[B57] Commons KG van Bockstaele EJ Pfaff DW Frequent colocalization of mu opioid and NMDA-type glutamate receptors at postsynaptic sites in periaqueductal gray neurons J Comp Neurol 1999 408 549 559 10.1002/(SICI)1096-9861(19990614)408:4<549::AID-CNE8>3.0.CO;2-3 10340504

[B58] Mao J Mayer DJ Spinal cord neuroplasticity following repeated opioid exposure and its relation to pathological pain Ann N Y Acad Sci 2001 933 175 184 12000019 10.1111/j.1749-6632.2001.tb05823.x

[B59] Taddese A Nah SY McCleskey EW Selective opioid inhibition of small nociceptive neurons Science 1995 270 1366 1369 10.1126/science.270.5240.1366 7481826

[B60] Nishizuka Y Intracellular signaling by hydrolysis of phospholipids and activation of protein kinase C Science 1992 258 607 614 10.1126/science.1411571 1411571

[B61] Fan GH Zhao J Wu YL Lou LG Zhang Z Jing Q Ma L Pei G N-Methyl-D-aspartate attenuates opioid receptor-mediated G protein activation and this process involves protein kinase C Mol Pharmacol 1998 53 684 690 9547359 10.1124/mol.53.4.684

[B62] Kieffer BL Evans CJ Opioid tolerance-in search of the holy grail Cell 2002 108 587 590 10.1016/S0092-8674(02)00666-9 11893329

[B63] Nicoll RA Malenka RC Expression mechanisms underlying NMDA receptor-dependent long-term potentiation Ann N Y Acad Sci 1999 868 515 525 10.1111/j.1749-6632.1999.tb11320.x 10414328

[B64] Ruscheweyh R Ikeda H Heinke B Sandkuhler J Distinctive membrane and discharge properties of rat spinal lamina I projection neurones in vitro J Physiol 2004 555 527 543 14694142 10.1113/jphysiol.2003.054049 PMC1664848

[B65] Ueda H Inoue M Mizuno K New approaches to study the development of morphine tolerance and dependence Life Sci 2003 74 313 320 10.1016/j.lfs.2003.09.018 14607259

[B66] Menard DP van Rossum D Kar S Jolicoeur FB Jhamandas K Quirion R Tolerance to the antinociceptive properties of morphine in the rat spinal cord: alteration of calcitonin gene-related peptide-like immunostaining and receptor binding sites J Pharmacol Exp Ther 1995 273 887 894 7752094

[B67] Powell KJ Quirion R Jhamandas K Inhibition of neurokinin-1-substance P receptor and prostanoid activity prevents and reverses the development of morphine tolerance in vivo and the morphine-induced increase in CGRP expression in cultured dorsal root ganglion neurons Eur J Neurosci 2003 18 1572 1583 10.1046/j.1460-9568.2003.02887.x 14511336

[B68] Zhu H Barr GA Opiate withdrawal during development: are NMDA receptors indispensable? Trends Pharmacol Sci 2001 22 404 408 10.1016/S0165-6147(00)01792-2 11479002

[B69] Hoskins B Ho IK Meydrech EF Effects of aging and morphine administration on calmodulin and calmodulin-regulated enzymes in striata of mice J Neurochem 1985 44 1069 1073 10.1111/j.1471-4159.1985.tb08726.x 2857771

[B70] Pascale A Govoni S Battaini F Age-related alteration of PKC, a key enzyme in memory processes: physiological and pathological examples Mol Neurobiol 1998 16 49 62 10.1007/BF02740602 9554701

[B71] Ross EM Wilkie TM GTPase-activating proteins for heterotrimeric G proteins: regulators of G protein signaling (RGS) and RGS-like proteins Annu Rev Biochem 2000 69 795 827 10.1146/annurev.biochem.69.1.795 10966476

[B72] Xie GX Palmer PP RGS proteins: new players in the field of opioid signaling and tolerance mechanisms Anesth Analg 2005 100 1034 1042 10.1213/01.ANE.0000147711.51122.4B 15781518

[B73] Singleton MA Rosen JI Fisher DM Pharmacokinetics of fentanyl in the elderly Br J Anaesth 1988 60 619 622 10.1093/bja/60.6.619 3377944

[B74] Ihnatovych I Novotny J Haugvicova R Bourova L Mares P Svoboda P Ontogenetic development of the G protein-mediated adenylyl cyclase signalling in rat brain Brain Res Dev Brain Res 2002 133 69 75 10.1016/s0165-3806(01)00323-6 11850065

[B75] Burton CK Ho IK Hoskins B Evidence for involvement of cyclic GMP phosphodiesterase in morphine tolerance J Pharmacol Exp Ther 1990 252 104 111 2153795

[B76] Grillet N Dubreuil V Dufour HD Brunet JF Dynamic expression of RGS4 in the developing nervous system and regulation by the neural type-specific transcription factor Phox2b J Neurosci 2003 23 10613 10621 14627646 10.1523/JNEUROSCI.23-33-10613.2003 PMC6740911

[B77] Ingi T Aoki Y Expression of RGS2, RGS4 and RGS7 in the developing postnatal brain Eur J Neurosci 2002 15 929 936 10.1046/j.1460-9568.2002.01925.x 11906535

[B78] Gold SJ Ni YG Dohlman HG Nestler EJ Regulators of G-protein signaling (RGS) proteins: region-specific expression of nine subtypes in rat brain J Neurosci 1997 17 8024 8037 9315921 10.1523/JNEUROSCI.17-20-08024.1997 PMC6793903

[B79] Wilson LD Ross SA Lepore DA Wada T Penninger JM Thomas PQ Developmentally regulated expression of the regulator of G-protein signaling gene 2 (Rgs2) in the embryonic mouse pituitary Gene Expr Patterns 2005 5 305 311 10.1016/j.modgep.2004.10.005 15661635

[B80] Thomas EA Danielson PE Sutcliffe JG RGS9: a regulator of G-protein signalling with specific expression in rat and mouse striatum J Neurosci Res 1998 52 118 124 10.1002/(SICI)1097-4547(19980401)52:1<118::AID-JNR11>3.0.CO;2-6 9556034

[B81] Kim KJ Moriyama K Han KR Sharma M Han X Xie GX Palmer PP Differential expression of the regulator of G protein signaling RGS9 protein in nociceptive pathways of different age rats Brain Res Dev Brain Res 2005 160 28 39 10.1016/j.devbrainres.2005.08.003 16153714

[B82] Stohr J Bourova L Hejnova L Ihnatovych I Novotny J Svoboda P Increased baclofen-stimulated G protein coupling and deactivation in rat brain cortex during development Brain Res Dev Brain Res 2004 151 67 73 10.1016/j.devbrainres.2004.03.014 15246693

[B83] Yu JH Wieser J Adams TH The Aspergillus FlbA RGS domain protein antagonizes G protein signaling to block proliferation and allow development EMBO J 1996 15 5184 5190 8895563 PMC452262

[B84] Ingi T Krumins AM Chidiac P Brothers GM Chung S Snow BE Barnes CA Lanahan AA Siderovski DP Ross EM Gilman AG Worley PF Dynamic regulation of RGS2 suggests a novel mechanism in G-protein signaling and neuronal plasticity J Neurosci 1998 18 7178 7188 9736641 10.1523/JNEUROSCI.18-18-07178.1998 PMC6793237

[B85] Granderath S Stollewerk A Greig S Goodman CS O'Kane CJ Klambt C loco encodes an RGS protein required for Drosophila glial differentiation Development 1999 126 1781 1791 10079238 10.1242/dev.126.8.1781

[B86] Wu C Zeng Q Blumer KJ Muslin AJ RGS proteins inhibit Xwnt-8 signaling in Xenopus embryonic development Development 2000 127 2773 2784 10851124 10.1242/dev.127.13.2773

[B87] Tekumalla PK Calon F Rahman Z Birdi S Rajput AH Hornykiewicz O Di Paolo T Bedard PJ Nestler EJ Elevated levels of DeltaFosB and RGS9 in striatum in Parkinson's disease Biol Psychiatry 2001 50 813 816 10.1016/S0006-3223(01)01234-3 11720701

[B88] Muma NA Mariyappa R Williams K Lee JM Differences in regional and subcellular localization of G(q/11) and RGS4 protein levels in Alzheimer's disease: correlation with muscarinic M1 receptor binding parameters Synapse 2003 47 58 65 10.1002/syn.10153 12422374

[B89] Van Crugten JT Somogyi AA Nation RL Reynolds G The effect of old age on the disposition and antinociceptive response of morphine and morphine-6 beta-glucuronide in the rat Pain 1997 71 199 205 10.1016/S0304-3959(97)03363-0 9211481

[B90] Minto CF Schnider TW Egan TD Youngs E Lemmens HJ Gambus PL Billard V Hoke JF Moore KH Hermann DJ Muir KT Mandema JW Shafer SL Influence of age and gender on the pharmacokinetics and pharmacodynamics of remifentanil. I. Model development Anesthesiology 1997 86 10 23 10.1097/00000542-199701000-00004 9009935

[B91] Bouwmeester NJ Anderson BJ Tibboel D Holford NH Developmental pharmacokinetics of morphine and its metabolites in neonates, infants and young children Br J Anaesth 2004 92 208 217 10.1093/bja/aeh042 14722170

[B92] Bhargava HN Villar VM Rahmani NH Larsen AK Studies on the possible role of pharmacokinetics in the development of tolerance to morphine in the rat Gen Pharmacol 1992 23 1199 1204 10.1016/0306-3623(92)90312-8 1487129

[B93] Gardell LR King T Ossipov MH Rice KC Lai J Vanderah TW Porreca F Opioid receptor-mediated hyperalgesia and antinociceptive tolerance induced by sustained opiate delivery Neurosci Lett 2006 396 44 49 10.1016/j.neulet.2005.11.009 16343768

